
JOURNAL INFORMATION
==============================
NLM Title Abbreviation: ScientificWorldJournal
Journal ID: ScientificWorldJournal
NLM Journal ID: 101131163
Journal ID: TSWJ

The Scientific World Journal

ISSN: 2356-6140
EISSN: 1537-744X
Publisher: Wiley

ARTICLE INFORMATION
==============================
PMCID: PMC6009259
PMID: 29973776
DOI: 10.1100/tsw.2002.2
Article ID: 296397
Article version: 1
Subjects: Short Report

The Mechanism of RNA Interference and the Transposon Silencing in Caenorhabditis elegans

Tijsterman Marcel
Ketting Rena
Fischer Sylvia
Simmer Femke
Sijen Titia
Okihara Kristy
Tops Bas
Vastenhouw Nadine
Plasterk Ronald *
Hubrecht Laboratory, Uppsalalaan 8, 3584 CT Utrecht, The Netherlands
*Ronald Plasterk: plasterk@niob.knaw.nl
Publication date: 2002
Electronic publication date: 2002 Jan 23
Volume: 2
First page: 3
Last page: 4
Copyright: Copyright © 2002 Marcel Tijsterman et al.
Copyright year: 2002
License: This is an open access article distributed under the Creative Commons Attribution License, which permits unrestricted use, distribution, and reproduction in any medium, provided the original work is properly cited.
License URL: https://creativecommons.org/licenses/by/3.0/

==============================
Advances in Gene Technology: The Genome and Beyond –
Structural Biology for Medicine (Proceedings of the 2002 Miami
*Nature Biotechnology* Winter Symposium)
*TheScientificWorld* 2002, 2(S2), 3–4
ISSN 1532-2246; DOI 10.1100/tsw.2002.2

### THE MECHANISM OF RNA INTERFERENCE AND THE TRANSPOSON SILENCING IN *CAENORHABDITIS ELEGANS*

Marcel Tijsterman, René Ketting, Sylvia Fischer, Femke Simmer, Titia Sijen, Kristy Okihara, Bas Tops, Nadine Vastenhouw, and Ronald Plasterk
Hubrecht Laboratory, Uppsalalaan 8, 3584 CT Utrecht, the Netherlands
plasterk@niob.knaw.nl

**INTRODUCTION.** All isolates of *C. elegans* contain multiple transposable elements in their genome. These elements jump around freely in somatic cells, but transposition is fully silenced in the germline. In investigating the mechanism of transposon silencing, we found that it was mechanistically linked to another phenomenon: RNAi or RNA interference. This is the experimental silencing of gene expression by administration of double-stranded RNA.

**METHOD.** Mutants have been isolated that were defective in transposon silencing and/or in RNA interference. Several of these mutants have now been identified at the molecular level, thus defining essential components of the silencing machinery. The first biochemical analysis of the mechanism of silencing has also been initiated.

**RESULTS.** Our current picture of the pathway of silencing is shown in Fig. 1. (Marcel) Several of the mutator proteins will be discussed in detail.

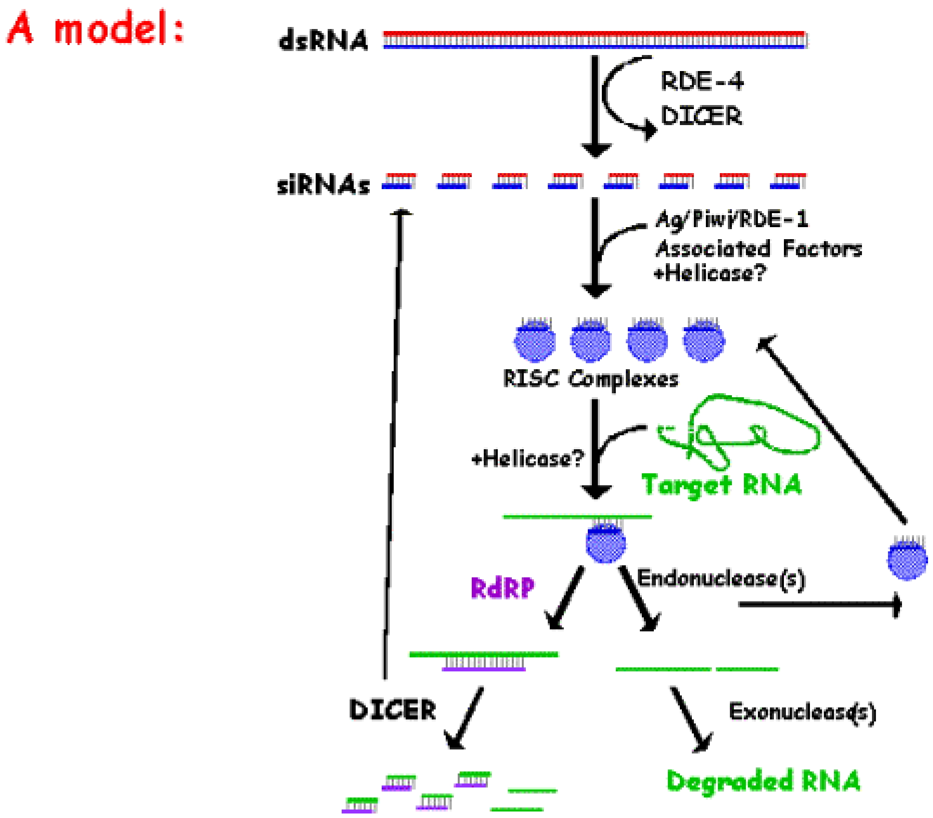

**DISCUSSION.** RNA interference is an experimental procedure that takes advantage of a mechanism that was probably meant for other purposes: the defense of the genome against the invasion of viruses and transposable elements. The human genome consists of approximately 50% repetitive DNA, derived from previous invasions by transposons and viruses. It is *a priori* to be expected that complex organisms must have 'virus protection software', to limit such invasions to a minimum. This software must somehow be able to recognize the difference between *self* and *nonself* at the molecular level.

### REFERENCES

1. Ketting, R.F., Haverkamp,T.H.A., van Luenen, H.G.A.M., and Plasterk, R.H.A. (1999) Cell 99, 133–141.
2. Ketting, R.F. and Plasterk, R.H.A. (2000) Nature 404, 296–298.
3. Ketting, R.F. and Plasterk, R.H.A. (2000) Curr. Opin. Genes Dev. 10, 562–567.
4. Ketting, R.F., Fischer, S.E.J., Bernstein, E., Sijen, T., Hannon, G.J., and Plasterk, R.H.A. (2001) Genes Dev. 15(20), in press.
